# Everybody's Page

**Published:** 1888-04-28

**Authors:** 


### Everybody's Page.

In London no street may be less than forty feet wide, exclusive of any gardens, open areas, fore-courts, or other spaces in front of the houses, and the height of a house must not exceed the width of the street.

There are well-authenticated instances of hospitals having a light and a dark side, in which the percentages of recovery have been distinctly greater in the former. In the Roosevelt hospital there is a glass case for a sun bath to hasten recovery, a revival of the solar bath of the ancient Romans.

Bright sunlight falling into a bedroom is very liable to awake those asleep in it, and as childen ought to have a large allowance of sleep, it is necessary in the summer time to exclude the sun's rays. in the early morning by shutters or otherwise.

A man who wilfully adulterates human food is worse than even the famous highwayman of old. These gentlemen of the road mercifully offered an alternative. "Your money or your life" was their demand; our modern food adulterators not only demand, but most surely take both our money and our lives.

Public analysts have some strange experiences. One in the south of Scotland declares that one shopkeeper knew him so well that he kept a special set of bags for his use; so that whether he bought sugar, tea, or oatmeal, it was always put into a bag bearing the inscription: "Notice.—This is sold as a mixture of chicory and coffee."

Mother-of-Pearl is not coloured, but the surface is covered with extremely fine grooves. The waves of light reflected from these grooves at different angles interfere with each other, so that some of the coloured waves of which white light is composed are extinguished, and those which are left impart the colour. The kind of wave extinguished depends upon the angle at which the light falls, therefore the play of colours varies with the change of position.

The chances of death are at their maximum immediately after birth. It has been shown that of all the children born, one-tenth die during the first month of life; that by the end of the first year only three-fourths are alive, and that in the towns nearly a half of the total number born die before the fifth year. As age advances, however, things get better, and about the eleventh or twelfth year the chances of death are least. After this, however, they increase, and go on increasing rapidly till about the twenty-fifth year, when they are a little less (in man) till the thirtieth or thirty-fifth year, when they gradually increase with age.

Pope said of vice that "grown at last familiar with her face, we first endure, then pity, then embrace." The same law seems to hold good of adulterated food. Many people think their coffee poor stuff if it is not flavoured with chicory, and now it appears that there are a number who prefer to have their butter adulterated with margarine. Most of the Normandy butter sent to this country is so doctored, and Mr. Bernal, the Consul-General at Havre, tells a sad story of an exporter of butter, who, having been in the habit of adding margarine to his butter, once in a fit of honesty sent a consignment to England pure. What was the result? The consignee wrote an indignant letter saying the last butter sent was of decidedly inferior quality. What was the Norman to do but fall back on the old plan? Vitiated tastes in the purchaser are a great hindrance to honesty in the vendor, let Acts of Parliament do what they will.

The risks to public health of over education are far less than those of under education. Hysteria, hypochondriasis, insanity, and nervous diseases spring from idleness and ignorance to a far greater extent than from over-pressure.

Attention to the skin is of the very first importance as a measure for enabling us to withstand the injurious effects of cold. It is chiefly by the skin that we catch a cold, and it is certainly by the skin that we may most successfully prevent a cold.

It is a curious point that the range of diversity of size in the same individual appears to be more marked in our own civilized heads than in certain less civilized races, such as the New Zealanders and South American Indians. This alone might be considered as indicating that we belong to a more mixed race.

We have been furnished by Messrs. John Dewar and Sons, of Perth, with a sample of special old Highland whisky. The whisky is well matured, of a fine soft taste, free from fusel oil, and exceedingly wholesome. For invalids requiring stimulants, and for those who, though not exactly invalids, find small quantities of alcohol beneficial, well-matured Scotch whiskies of the highest class are strongly to be recommended.

As civilization progresses, the number of deaths from paralysis will probably increase. This, however, is not a bad sign; it will show rather that longevity is increasing. Paralysis is distinctly a disease of old age, and its more frequent occurrence shows that those who die of it have survived the dangers which under less favourable sanitary conditions might have carried them off at an earlier period of life.

Milk is a sponge, and a dangerous sponge. It absorbs at once any deleterious matter, and is one of the most fertile causes of epidemics. In a paper by Mr. Hart, read before the Health Department of the Social Science Congress in Glasgow, the analyses 53 outbreaks of typhoid fever, 17 of scarlet fever, and 12 of diphtheria due to milk. The milk epidemics in question had attacked in round numbers 3,500 with typhoid, 880 with scarlet fever, and 700 with diphtheria. These cases have all occurred within the last twelve years.

The average thickness of a human hair taken from the head has been calculated by Sir Erasmus Wilson to be about $\frac{1}{400}$th of an inch. But the diameter of the hair is affected by many circumstances. We are in the habit of regarding man as being altogether a coarser animal than woman. This is not the case, however, in every particular, because female hair is, as a rule, not so fine as that of man, and this in spite of the frequent cutting to which the male hair is subjected. As might naturally be expected, the hair of the child is more delicate than that of a grown person.

Lady Ripon has opened her house for a series of lectures on the social position of women. Mrs. Henry Fawcett gave the first lecture last Friday afternoon to a large and appreciative audience. Miss Manning on "The Women of India," Mrs. Ayrton on "Women and Science," Mrs. Costelloe on "The Women of America," and Miss Amelia B. Edwards on the "Social and Political Position of Women in Ancient Egypt," will follow on the next four Fridays. The object is to raise funds for the St. Ursula Association, which "aims at bringing refining and elevating influences to bear upon those whose lives are dull and for the most part poverty-stricken"—namely, the factory and workshop girls, with whose trials and difficulties Mr. Besant has made us so well acquainted.

